# Increased Plasma Circulating Cell-Free DNA Could Be a Potential Marker for Oral Cancer

**DOI:** 10.3390/ijms19113303

**Published:** 2018-10-24

**Authors:** Li-Han Lin, Kuo-Wei Chang, Shou-Yen Kao, Hui-Wen Cheng, Chung-Ji Liu

**Affiliations:** 1 Department of Medical Research, MacKay Memorial Hospital, Taipei 10449, Taiwan; volkdeskkimo@gmail.com (L.-H.L.); amycheng.b784@mmh.org.tw (H.-W.C.); 2 Institute of Oral Biology, School of Dentistry, National Yang-Ming University, Department of Stomatology, Medical Education and Research, Veterans General Hospital, Taipei 11217, Taiwan; 4kuowei@gmail.com (K.-W.C.); sykao@vghtpe.gov.tw (S.-Y.K.); 3 Institute of Oral Biology, School of Dentistry, National Yang-Ming University, Department of Oral and Maxillofacial Surgery, Taipei MacKay Memorial Hospital, Taipei 10449, Taiwan

**Keywords:** cell free DNA, marker, mouth neoplasm, plasma, survival

## Abstract

Background: Oral squamous cell carcinoma (OSCC) is a disease that affects patients worldwide. DNA of dead cells is released into the blood stream and may be isolated from plasma or serum samples. This DNA is termed cell-free DNA (cfDNA). cfDNA is increased in several types of malignancies. We investigated if there was a correlation between cfDNA levels and the progression of OSCC. Methods: Using quantitative spectrometry, we measured plasma cfDNA in 121 patients with OSCC and 50 matched controls. Mann Whitney and Wilcoxon tests were used to compare differences among various clinical variants. Receiver operating characteristic (ROC) analysis was used to obtain levels suitable for the separation of the clinical subsets. Kaplan-Meier analysis was used to assess correlation with survival. Results: Plasma cfDNA was significantly elevated in patients with OSCC relative to controls. Plasma cfDNA levels correlated with larger tumor size, cervical lymph node metastasis and late stage. Higher plasma cfDNA levels were associated with a poor prognosis of OSCC, which is a new finding. Conclusion: Plasma cfDNA could serve as a novel and easily accessible biomarker in OSCC, providing diagnostic and prognostic value.

## 1. Introduction

Oral squamous cell carcinoma (OSCC) is one of the most frequent carcinomas worldwide [1,2,3,4,5] and usually is the result of a carcinogenic process which is comprising several steps. In addition, multiple lesions (which may be at different stages of neoplasm) may develop at the same time and over large mucosal areas and turn into cancers. This may be the reason for the high rate of recurrence of OSCC after treatment [6]. Therefore, in order to improve the diagnosis of individuals at risk as well as the treatment of patients, more biomarkers which are sensitive and specific for OSCC are highly sought [7].

Cell free DNA (cfDNA) can be derived from normal cells, including normal leukocytes that undergo apoptosis, be shed DNA from dead healthy cells or from cancer cells; it is thus detectable in healthy people, patients without cancer, patients with benign tumors and in cancer patients [8,9]. Total cfDNA thus is the sum of normal and tumor cfDNA, with variable proportions [10]. cfDNA has recently received renewed interest for its potential use as a biomarker in oncology [11]. Over the last decade, several studies have demonstrated that cfDNA analysis is a source of information on tumor presence and molecular composition without the need for direct tumor biopsy [12,13]. In addition, cfDNA may originate from circulating tumor cells and thus may reflect micro-metastatic disease and aggressiveness of the disease [14]. In head and neck cancer, oropharyngeal squamous cell carcinoma (OPSCC) had been reported to show higher plasma cfDNA levels than other types of head and squamous cell carcinomas [15].

Several markers were found in OSCC blood samples [16,17,18,19]. In our previous studies, we used the serum levels of matrix metalloproteinase-9 (MMP-9), vascular endothelial growth factor (VEGF), microRNA-31(miR-31) and platelet count from patients to evaluate the prognosis of OSCC [4,20,21]. Recent studies have detected circulatory cfDNA in the plasma of patients with different types of cancer [22,23,24]. However, plasma cfDNA levels have not yet been associated with OSCC progression and prognosis in previous studies [25,26]. Though Mazurek et al. have reported elevated plasma cfDNA level in OPSCC compared to other types of HNSCC (there were no OSCC group), there was no statistical significance between the cancer and control groups in their study [15]. This investigation evaluated whether plasma cfDNA levels could be a potential non-invasive marker for OSCC.

## 2. Results

### 2.1. Patient Characteristics

**Figure 1:**
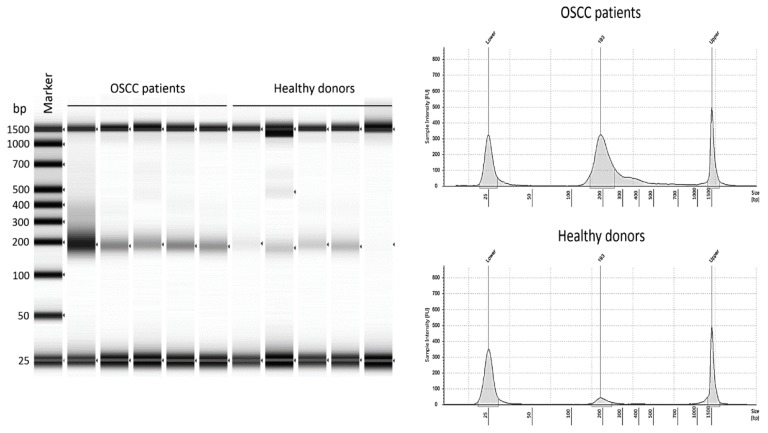
Agilent TapeStation analysis for the DNA integrity of oral squamous cell carcinoma (OSCC) samples and healthy donors.

In total, 121 patients with OSCC and 50 matched healthy controls were enrolled in this study. The size distribution of cfDNA was similar in OSCC and healthy donors, the average size was 150 to 200 bp (Figure 1). The clinical characteristics of our study subjects have been listed in Table 1.

### 2.2. Plasma cfDNA as a Potential Diagnostic Marker

**Figure 2:**
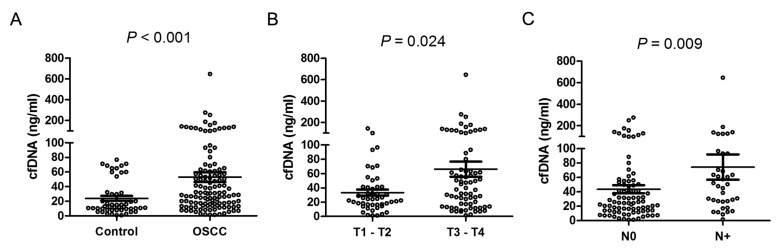

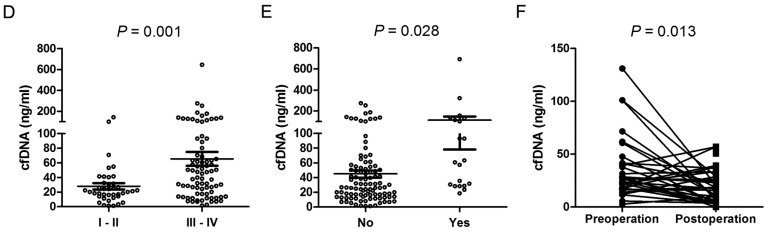
Comparison of cell free DNA (cfDNA) plasma levels. Scatter dot plots with mean ± SD computed by Mann–Whitney test: (**A**) Healthy controls vs. preoperative plasma cfDNA levels in patients with OSCC. (**B**) pre-operative plasma cfDNA levels and different tumor sizes (**C**), Absence or presence of neck lymph node metastasis. (**D**) early and late stage carcinoma and (**E**) lymphovascular invasion status. (**F**) pre-operative and post-operative plasma samples.

**Figure 3:**
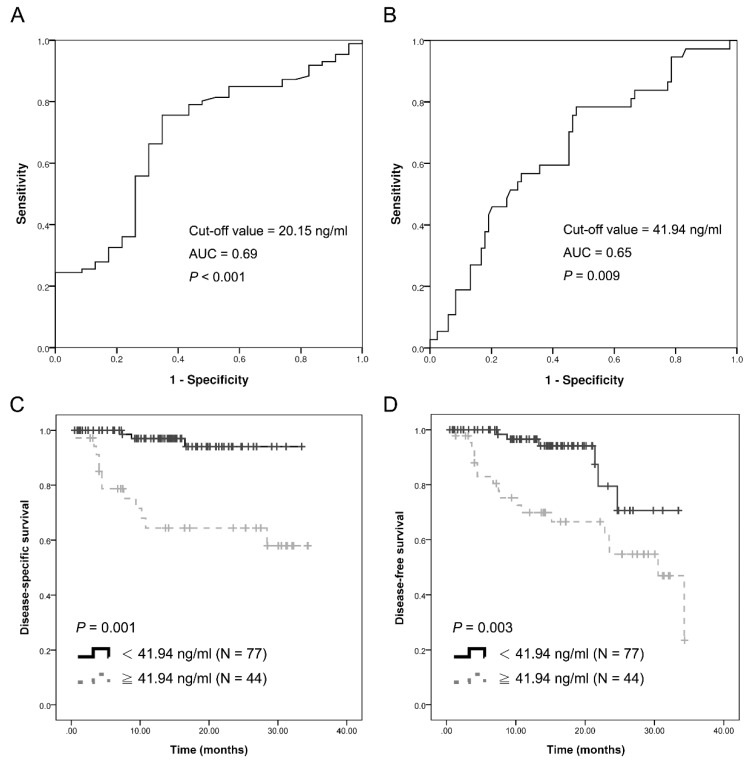
Receiver operating characteristic (ROC) and leave-one-out cross-validation (LOOCV) analysis across control and pre-operative samples (**A**) lymph node metastasis status (**B**) and disease specific survival samples (**C**). Plasma cfDNA Kaplan–Meier analysis of disease specific survival for OSCC patients. Higher plasma cfDNA concentrations demonstrated a poor prognosis in OSCC. (**D**). Plasma cfDNA Kaplan–Meier analysis of disease free survival for OSCC patients. Higher plasma cfDNA concentrations demonstrated more relapsed in OSCC.

Patients with OSCC had pre-operative cfDNA plasma concentrations ranging from 11.3 to 646 ng/mL which was significantly higher than in the control group (0.79 to 76.8 ng/mL, Figure 2A). The mean concentration of cfDNA in OSCC was 53.1 ± 6.69 ng/mL, as compared with 24.0 ± 3.33 ng/mL in the control group. When using 20.2 ng/mL as the cutoff, this marker yielded an AUC of 0.69 in receiver operating characteristic (ROC) and an accuracy of 0.68 as defined by the Leave-one-out cross-validation (LOOCV, Figure 3A). A multivariate logistic regression analysis indicated an adjusted odds ratio of 4.15 (95% CI, 2.16–9.20; *p* < 0.001, Table 2).

### 2.3. cfDNA Level as an Independent Factor of Cervical Lymph Node Metastasis in OSCC

Several clinical parameters were analyzed in this study. cfDNA was not associated with age, gender, perineural invasion and cell differentiation (Table 1). cfDNA levels however were related to tumor size (Figure 2B), TNM staging (Figure 2D) and lymphovascular invasion (Figure 2E). Higher plasma cfDNA levels were found in tumor patients with neck lymph node metastasis compared to those without these features (Figure 2C). When using 42.0 ng/mL as cutoff, this marker yielded an AUC of 0.65 and an accuracy of 0.60 in ROC analysis, as defined by LOOCV (Figure 3B). A multivariate logistic regression analysis indicated an adjusted odds ratio of 2.53 (95% CI, 1.06–6.08; *p* = 0.038, Table 3). 

### 2.4. Decrease of cfDNA in Patients’ Plasma after the Resection of Oral Primary Tumors

We further investigated if the levels of plasma cfDNA from patients with OSCC would change after ablative tumor surgery and found a significant decrease in cfDNA after tumor resection in 75% of the patients (45 of 60, Figure 2F). 

### 2.5. Association between cfDNA Levels and Survival of Patients with OSCC

There were several clinical parameters associated with disease specific survival in OSCC, including tumor size, node stage, perineural invasion, lymphovascular invasion and cfDNA levels in a univariate analysis (Table 4). When adjusting for tumor size, perineural invasion and lymphovascular invasion, neck lymph node metastasis (hazard ratio, 9.529; 95% CI, 2.054 to 44.195; *p* = 0.004) and cfDNA level (hazard ratio, 4.432; 95% CI, 1.214 to 16.178; *p* = 0.024) were independent factors influencing disease specific survival (Table 4). Kaplan–Meier analysis indicated an association of higher cfDNA levels with worse disease-specific survival (*p* = 0.001) and disease-free survival (*p* = 0.003) (Figure 3C,D).

## 3. Discussion

cfDNA was discovered in 1948 by Mandel and Metais [27] but there was no interest in cfDNA until 40 years thereafter. Research on cfDNA in the human circulatory system has been conducted in various clinical fields. Leon [28] demonstrated that the serum cfDNA concentration was significantly increased in cancer patients. Stroun et al. detected cfDNA in 27% of a cancer patient group while it was absent in healthy controls, suggesting a correlation with malignancy [29,30]. Coulet et al. used fluorescence emission after adding a dye intercalating with Plasma DNA to test for circulating DNA in HNSCC. Plasma DNA concentrations however did not significantly correlate between gender, tumor stage or tumor localization [26]. In the recent years, Shukla et al. found that cfDNA values in the plasma and blood were not significantly elevated in patients with precancerous lesions, patients with OSCC, or patients with OSCC after surgery, as compared with healthy subjects [25]. However, in the majority of studies, cfDNA has been reported to be a highly sensitive genetic biomarker in several types of cancer which directly reflected the tumor burden and genetic dynamics, including pancreatic [10], melanoma [31], lung [13,22,32,33], colorectal [34], breast [35,36] and prostate cancer. In recently Desai, reported total cfDNA may be applied as a screening marker for early detection of pre-cancer and cancer as well as for prognostication of oral cancer [37]. Only forty OSCC samples enrolled in this study, clinicopathological parameters cannot analyze via the limited samples size. In our study, we measured the OSCC plasma cfDNA concentration. Our results demonstrate that plasma cfDNA levels are higher in patients with OSCC, compared to healthy controls. This is different to a previous study of OSCC [25], this result may be similar to other types of solid malignancies [38,39].

There are two common methods to characterize cfDNA: First, the quantification of total cfDNA using for example, spectrophotometry and, second, the detection of specific tumor markers by for example, polymerase chain reaction (PCR)–based techniques along with sequencing. Both strategies are effective for detecting cfDNA but the combined hazard ratios seemed to be more prominent in subgroups where specific tumor markers were assayed, rather than in those where cfDNA was quantified [34]. However, most manufacturers of real-time quantitative PCR (qPCR) instruments and qPCR reagents recommend amplicon lengths of 80–150 bp, as longer products have lowered amplification efficiencies, which may increase variation and thus decrease the reliability of the results [40,41]. It should be noted that the cfDNA quantities based on the measurement of some target genes (e.g., human telomerase reverse transcriptase TERT) were more than several folds higher than those of other assays [15]. However, debates are continuing regarding accuracy and sensitivity [42]. In this study, we used a spectrophotometric analyzer, the TapeStation 2200 (Agilent Technology, Santa Clara, CA, USA), equipped with the highly senitiveD1000 ScreenTape system (Agilent Technologies, Santa Clara, CA, USA) to measure small double-stranded DNA. The highly sensitivity D1000 ScreenTape system is designed for analyzing DNA molecules from 35 to 1000 bp. With a quantitative range of 10–1000 ng/mL and sensitivity of 5 ng/mL The Agilent 2200 TapeStation system in conjunction with the Agilent Genomic DNA ScreenTape assay provides an excellent solution for assessing the quantity, integrity and overall quality of genomic DNA. The quantification of the TapeStation 2200 is highly comparable to the fluorescence-based Qubit instrument. Differences seen with UV based detection are attributed to the differences in the measurement method between the fluorescence based platforms and the spectrophotometric NanoDrop [43,44]. This rationale may explain why our cfDNA sensitivity was higher than those previously reported.

While investigating the total amount of cfDNA in patients with malignant diseases, the risk of lysis because of prolonged stasis during blood sampling is an important factor to avoid, as it may influence the results [45]. Accurate quantification of low occurrence targets means that any release of genomic DNA (gDNA) from white blood cells (WBCs) following a blood draw should be minimized during sample storage and shipping so the proportion of specific cfDNA targets is preserved [46]. Obscuring cfDNA with gDNA could hamper detection in downstream applications [47]. Alterations in the ratio of cfDNA and gDNA might impair detection [46,47]. Thus, in order to be able to measure targets which are present in low concentrations only, the blood samples need to be handled carefully after drawing, during storage and during shipping, as the potential release of genomic DNA (gDNA) from white blood cells (WBCs) might obscure the cfDNA, which are specific for the investigated targets. We therefore immediately processed the blood after venous puncture by centrifuging the blood in a way that prevents the release of gDNA into the plasma fraction [48]. Additional studies have shown that true cfDNA fragments are generally <200 bp and are likely due to cellular apoptosis [49]. Increases in the concentration of fragments of >300 bp may be an indication of a compromised blood sample in which nucleated cells have released gDNA; Li and colleagues demonstrated that the majority of circulating DNA was <313 bp [50]. Therefore, having the ability to assess the degree of gDNA contamination in plasma may be useful in determining sample quality and integrity. True cfDNA fragments have been shown to be shorter than 200 bp in size and to originate from cellular apoptosis [49], whereas an increase in the concentration of fragments larger than 300 bp might pinpoint compromised blood samples where nucleated cells have released gDNA. Li and colleagues found that the majority of circulating DNA is below 313 bp in size [50]. Thus, being able to measure the amount of contamination by gDNA is helpful in assessing the quality and integrity of a sample. The highly sensitive D1000 ScreenTape system (Agilent Technologies, Santa Clara, CA, USA) may easily assess the integrity of double-stranded DNA and the size of cfDNA. We excluded eleven samples (seven from patients and four from controls) that were contaminated with a large amount of cfDNA, in order to ensure accuracy of our data. 

The mean concentration of cfDNA in the OSCC samples obtained using this method was 53.06 ng/mL. Compared with lung cancer, the mean plasma cfDNA concentration ranged from 29.5 to 270.0 ng/mL [22,39,51,52]. A previous study in OPSCC had a mean plasma concentration of 9.60 ng/mL [15]. Such differences in absolute concentration may come from blood sample processing, plasma isolation and storage or DNA quantification methods, as well as from the heterogeneity of the population studied and different cancer types. Regarding the potential impact of sample conservation time (long-term freezing may alter the quality and quantity of cfDNA) [22,28,42]. Several factors: The processing of blood samples, that is, the isolation of plasma, the way of storing it and the method of quantifying the DNA may contribute to the level of variation of cfDNA, in addition to the heterogeneity of the patient group under investigation and different cancer types as source material. The duration of storage until analysis also may play an important role, because also long-term freezing is known to alter the quality and quantity of cfDNA [22,28,42]. Thus, in this study, for all samples, plasma isolation was performed immediately after blood collection and the same standard procedures were used to limit possible contamination with blood cell DNA. Furthermore, cfDNA extraction and quantification were centrally performed in one laboratory using a standard protocol in order to limit quantification bias. 

There is some controversy regarding the relationship between cfDNA levels and clinicopathological features [53]. In OPSCC, increased plasma cfDNA levels were found in patients with clinical N2-N3 lymph node metastases but not in clinical N0-N1 patients [15]. Highly significant differences were found by considering T stage in patients who had cancer and had positive regional lymph node metastasis and significantly higher cfDNA levels were found in breast cancer [54]. cfDNA is not specific to neoplastic conditions. It may also be elevated in inflammatory and infectious conditions. Thus, the association between high cfDNA concentration and poor prognosis may be linked to tumor burden and/or comorbidities [55]. The association between high levels of cfDNA concentration and poor prognosis may be linked to the tumor burden and/or comorbidities, as cfDNA is not specific to neoplasms. It also may be elevated as a consequence of inflammation and infection [55]. Our study showed that there was a high level of plasma cfDNA in patients with OSCC who had large tumors, cervical lymph node metastasis and TNM staging. cfDNA is an independent indicator of cervical lymph node metastasis. The role of cfDNA is not yet entirely understood but plasma containing cfDNA has been demonstrated to transfect cells in vitro and in vivo [15]. Reporter gene was found in tissues of rats injected with plasma from tumor-bearing animals in experiments by Garcia-Olmo et al. [56]. As the circulating in the plasma and oncogenes that are derived from the primary tumor, the metastasis might occur in the transfection of susceptible cells located in distant target organs. Apart from the transformation function of cfDNA, it can also be used for the communication between cells [57]. which are hitherto not yet fully understood: Plasma containing cfDNA has been reported to transfect cells in vitro as well as in vivo [15] and Garcia-Olmo et al. have detected reporter genes expressed in tissues of rats after injecting plasma from tumor bearing animals [56]. This suggests the possibility that metastasis in distant organs is driven by circulating ongogenes which have been released by a primary tumor. Besides this, cfDNA also may play a role in intercellular communication [57]. We analyzed the overall amount of cfDNA comprehensively. Its overall concentration may reflect the proportion of cancer derivatives in circulation and may better represent tumor dynamics and reduce bias compared to single-site biopsies [58,59]. 

Our multivariate analysis suggests the association of cfDNA with a significantly worse disease specific survival. High levels of total cfDNA and the presence of cfDNA are associated with a lower survival rate in several solid tumors [34,38]. In our data, higher plasma cfDNA concentrations were an independent factor for disease specific survival in OSCC. This is presented in the literature for the first time. 

As thus far, no specific plasma marker for OSCC exists, it will be important to investigate on a broader basis if the measurement of cfDNA concentrations in plasma samples can provide significant information on the risk of having OSCC [7]. Despite that the quantitative methodologies proposed in the present study could still be immature for routine clinical uses as restricted by cost and technical feasibility, the design of rapid and convenient practices will improve OSCC diagnosis.

The methods and strategies which have been used in this study still are not optimal to be used in the clinical routine, due to high costs and a rather complex technical process. However, one should endeavor to develop simple and rapid analytics of cfDNA in order to improve diagnosis for the benefit of OSCC patients.

## 4. Materials and Methods

### 4.1. Plasma Samples

The blood samples were collected from 121 patients with OSCC prior to definite surgical excision. Patients underwent the operation from February 2014 to December 2015 (Table 1) and provided written informed consent. Patients were enrolled in this study if the following inclusion criteria were met: (1) histological diagnosis of squamous cell carcinoma and (2) definitive surgical intervention as the initial treatment modality. Exclusion criteria were: (1) recurrent or metastatic disease, (2) previous treatment with radiation or chemotherapy and (3) a history of synchronous or metachronous cancers. Tumor staging was performed according to the American Joint Committee on Cancer (AJCC 7th edition) guidelines for tumor, node and metastasis (TNM) classification. Patients with positive cervical lymph nodes, perineural invasion, lymphovascular invasion and closed margins were administered postoperative adjuvant concurrent chemotherapy/radiation treatment according to the National Comprehensive Cancer Network (NCCN) guidelines. From 60 patients, additional samples were obtained six months after the initial surgical treatment. Fifty age, gender and oral habit matched healthy people who underwent routine dental checkup were enrolled as healthy controls. These samples were collected after obtaining written informed consent. This study was approved by the Institutional Review Board (IRB) of Mackay Memorial Hospital, Taipei, in 27/01/2016, IRB project identification code number 15MMHIS104.

Ten milliliters of whole blood were collected from each individual in the morning after fasting using vacutainer tubes containing ethylenediaminetetraacetic acid (EDTA) as an anticoagulant (Becton Dickson, Franklin Lakes, NJ, USA). Patients were followed for an average period of about 28 months. 

### 4.2. cfDNA Extraction

Procedures for cfDNA extraction were performed as previously described [60]. The plasma samples were prepared by centrifuging the blood at 1600× *g* for 15 min, followed by centrifugation of the supernatant at 1600× *g* for 15 min. Then, the plasma was stored in 1 mL aliquots at −80 °C until analysis. The separation and storage of the plasma samples was performed within 3 h of blood collection at 4 °C. cfDNA was extracted from 1 mL plasma aliquots using the QIAamp Circulating Nucleic Acid Kit (QIAamp-Blood Mini Kit, QIAGEN, Chatsworth, CA, USA) following the manufacturer’s recommendations and was eluted in 60 μL elution buffer from the kit.

### 4.3. Plasma DNA Quantification 

Purified plasma DNA was quantified using a TapeStation 2200 (Agilent Technology, Santa Clara, CA, USA) with a high-sensitivity D1000 ScreenTape system (Agilent Technologies, Santa Clara, CA, USA). The system can analyze up to 96 samples per run and between 35 and 1000 bp. The assay is suited for accurate sizing and quantification of DNA fragments in high-throughput applications [61].

### 4.4. Statistical Analysis

Each analysis was performed using the Prism 5 statistical software program package (GraphPad, San Diego, CA, USA) and SPSS 18.0 (SPSS Inc., Chicago, IL, USA). To compare differences among diverse clinical variants, Mann–Whitney, Wilcoxon matched-pairs and Kruskal-Wallis tests were used. A binary logistic regression analysis was used to determine adjusted odds ratios and 95% confidence intervals (CI). A *p* value of <0.05 was recognized as statistical significance. By using receiver operating characteristic (ROC) analysis, different clinical subsets can be efficiently separated by the obtained level; the under-curve area (AUC) was used to test discriminative ability. A Kaplan–Meier analysis was used to evaluate the influence on disease specific survival and disease-free survival. The relevance of nonreactive variables and disease-specific survival was assessed using a multivariate Cox proportional hazards model. Differences were statistically significant as any of the following conditions: * *p* < 0.05, ** *p* < 0.01, *** *p* < 0.001. Cross-comparisons with no significance were not marked.

Receiver operating characteristic (ROC) analysis was employed to determine if the clinical subsets could be effectively separated, the area under the curve (AUC) was used as measure for the discriminative ability. Influence on survival was assessed by Kaplan-Meier analysis. A multivariate Cox proportional hazards model was used to test the association of nonreactive variables with disease specific survival. Criteria for the statistical significance of differences were * *p* < 0.05, ** *p* < 0.01 and *** *p* < 0.001. Cross-comparisons which were not statistically significant have not been marked. 

## 5. Conclusions

In conclusion, our data suggest that the quantification of cfDNA from plasma may be a useful noninvasive technique in clinical practice. Our data show that the quantification of cfDNA from plasma samples may benefit the diagnosis and treatment of OSCC patients, as in OSCC, higher preoperative plasma cfDNA concentrations are independently associated with neck lymph node metastasis and poor prognosis. 

We therefore would like to suggest a large scale prospective clinical study to further and better assess the predictive value of plasma cfDNA for the detection, diagnosis, treatment and prognosis of OSCC in the population. 

## Figures and Tables

**Table 1:** Clinical parameter and cfDNA content in OSCC patients.

	OSCC					Control			
Variables	N	Mean	±SEM	Pa	Pb	N	Mean	±SEM	Pa
Age (years)									
<58	56	43.25	±6.31	0.385		24	27.88	±5.51	0.938
≥58	65	61.24	±11.03			26	20.35	±3.88	
Gender									
Male	113	53.90	±7.10	0.851		43	22.64	±3.35	0.935
Female	8	41.25	±13.71			7	32.04	±12.36	
Tumor size									
T1–T2	48	33.24	±4.24	0.024 *					
T3–T4	73	66.09	±10.49						
Nodal stage									
N0	84	43.73	±5.59	0.009 *					
N+	37	74.25	±17.52						
Stage									
I–II	40	27.95	±4.30	0.001 *					
III–IV	81	65.46	±9.49						
Histological grade									
Well	88	50.96	±5.32		0.268				
Moderate	25	69.87	±26.53						
Poor	8	23.61	±6.30						
Perineural invasion									
No	84	51.72	±8.92	0.269					
Yes	37	56.11	±8.48						
Lymphovascular invasion									
No	101	45.08	±4.96	0.028 *					
Yes	20	93.39	±30.92						

Pa, *p*-value by Mann-Whitney U test; Pb, *p*-value by Kruskal-Wallis test. * Statistically significant (*p* < 0.05).

**Table 2:** Association between cfDNA content and OSCC risk.

Variables	Cases (%)		Controls (%)		Crude OR (95% CI)	*p*	Adjusted ^a^ OR (95% CI)	*p*
cfDNA (ng/mL)								
<20.15	43	(35.5%)	35	(70.0%)	Reference		Reference	
≥20.15	78	(64.5%)	15	(30.0%)	4.233 (2.080–8.611)	<0.001 *	4.153 (2.155–9.204)	<0.001 *

^a^ Odds ratios were adjusted for age and sex OR, Odds ratio; CI, confidence interval, * *p* < 0.05.

**Table 3:** Univariate and multivariate analysis of risk factors for lymph node metastasis.

Variables	Subgroups	Odd Ratio (95% CI)	*p*
Univariate analysis			
Age (years)	≥58 vs. <58	0.746 (0.343–1.619)	0.458
Gender	Female vs. Male	1.394 (0.315–6.166)	0.661
Tumor size	T3–T4 vs. T1–T2	3.295 (1.350–8.042)	0.009 *
Perineural invasion	Yes vs. No	2.720 (0.889–6.373)	0.017 *
Lymphovascular invasion	Yes vs. No	5.958 (2.134–16.636)	0.001 *
cfDNA (ng/mL)	≥41.94 vs. <41.94	3.481 (1.551–7.810)	0.002 *
Multivariate analysis			
Tumor size	T3–T4 vs. T1–T2	2.059 (0.783–5.412)	0.143
Perineural invasion	Yes vs. No	1.537 (0.581–4.066)	0.387
Lymphovascular invasion	Yes vs. No	3.424 (1.065–11.010)	0.039 *
cfDNA (ng/mL)	≥41.94 vs. <41.94	2.533 (1.055–6.084)	0.038 *

CI, confidence interval. * *p* < 0.05.

**Table 4:** Univariate and multivariate Cox regression analysis of disease specific survival in OSCC.

Variables	Subgroups	Hazard Ratio (95%CI)	*p*
Univariate analysis			
Age (years)	≥58 vs. <58	1.438 (0.509–4.060)	0.493
Gender	Female vs. Male	1.073 (0.141–8.165)	0.946
Tumor size	T3–T4 vs. T1–T2	4.474 (1.008–19.850)	0.049 *
Nodal stage	N+ vs. N0	15.992 (3.601–71.030)	<0.001 *
Perineural invasion	Yes vs. No	4.871 (1.663–14.268)	0.004 *
Lymphovascular invasion	Yes vs. No	6.690 (2.365–18.926)	<0.001 *
cfDNA (ng/mL)	≥41.94 vs. <41.94	6.637 (1.859–23.702)	0.004 *
Multivariate analysis			
Tumor size	T3–T4 vs. T1–T2	2.937 (0.640–13.472)	0.166
Nodal stage	N+ vs. N0	9.529 (2.054–44.195)	0.004 *
Perineural invasion	Yes vs. No	2.747 (0.877–8.602)	0.083
Lymphovascular invasion	Yes vs. No	1.886 (0.625–5.685)	0.260
cfDNA (ng/mL)	≥41.94 vs. <41.94	4.432 (1.214–16.178)	0.024 *

CI, confidence interval. * *p* < 0.05.

## References

[1] Shieh T.M., Lin S.C., Liu C.J., Chang S.S., Ku T.H., Chang K.W. (2007). Association of expression aberrances and genetic polymorphisms of lysyl oxidase with areca-associated oral tumorigenesis. Clin. Cancer Res..

[2] Lin S.C., Liu C.J., Ko S.Y., Chang H.C., Liu T.Y., Chang K.W. (2005). Copy number amplification of 3q26-27 oncogenes in microdissected oral squamous cell carcinoma and oral brushed samples from areca chewers. J. Pathol..

[3] Chang K.W., Liu C.J., Chu T.H., Cheng H.W., Hung P.S., Hu W.Y., Lin S.C. (2008). Association between high miR-211 microRNA expression and the poor prognosis of oral carcinoma. J. Dent. Res..

[4] Liu C.J., Chang K.W., Lin S.C., Cheng H.W. (2009). Presurgical serum levels of matrix metalloproteinase-9 and vascular endothelial growth factor in oral squamous cell carcinoma. Oral Oncol..

[5] Wong T.S., Liu X.B., Wong B.Y., Ng R.W., Yuen A.P., Wei W.I. (2008). Mature miR-184 as Potential Oncogenic microRNA of Squamous Cell Carcinoma of Tongue. Clin. Cancer Res..

[6] Braakhuis B.J., Tabor M.P., Leemans C.R., van der Waal I., Snow G.B., Brakenhoff R.H. (2002). Second primary tumors and field cancerization in oral and oropharyngeal cancer: Molecular techniques provide new insights and definitions. Head Neck.

[7] Schmidt H., Kulasinghe A., Kenny L., Punyadeera C. (2016). The development of a liquid biopsy for head and neck cancers. Oral Oncol..

[8] Kidess E., Jeffrey S.S. (2013). Circulating tumor cells versus tumor-derived cell-free DNA: Rivals or partners in cancer care in the era of single-cell analysis?. Genome Med..

[9] Spellman P.T., Gray J.W. (2014). Detecting cancer by monitoring circulating tumor DNA. Nat. Med..

[10] Bettegowda C., Sausen M., Leary R.J., Kinde I., Wang Y., Agrawal N., Bartlett B.R., Wang H., Luber B., Alani R.M. (2014). Detection of circulating tumor DNA in early- and late-stage human malignancies. Sci. Transl. Med..

[11] Ulivi P., Silvestrini R. (2013). Role of quantitative and qualitative characteristics of free circulating DNA in the management of patients with non-small cell lung cancer. Cell. Oncol..

[12] Sidransky D. (2002). Emerging molecular markers of cancer. Nat. Rev. Cancer.

[13] Couraud S., Vaca-Paniagua F., Villar S., Oliver J., Schuster T., Blanche H., Girard N., Tredaniel J., Guilleminault L., Gervais R. (2014). Noninvasive diagnosis of actionable mutations by deep sequencing of circulating free DNA in lung cancer from never-smokers: A proof-of-concept study from BioCAST/IFCT-1002. Clin. Cancer Res..

[14] Jung K., Fleischhacker M., Rabien A. (2010). Cell-free DNA in the blood as a solid tumor biomarker--a critical appraisal of the literature. Clin. Chim. Acta.

[15] Mazurek A.M., Rutkowski T., Fiszer-Kierzkowska A., Malusecka E., Skladowski K. (2016). Assessment of the total cfDNA and HPV16/18 detection in plasma samples of head and neck squamous cell carcinoma patients. Oral Oncol..

[16] Cheng A.J., Chen L.C., Chien K.Y., Chen Y.J., Chang J.T., Wang H.M., Liao C.T., Chen I.H. (2005). Oral cancer plasma tumor marker identified with bead-based affinity-fractionated proteomic technology. Clin. Chem..

[17] Nakamoto D., Yamamoto N., Takagi R., Katakura A., Mizoe J.E., Shibahara T. (2006). Detection of tumor DNA in plasma using whole genome amplification. Bull. Tokyo Dent. Coll..

[18] Garcia-Olmo D.C., Gutierrez-Gonzalez L., Samos J., Picazo M.G., Atienzar M., Garcia-Olmo D. (2006). Surgery and hematogenous dissemination: Comparison between the detection of circulating tumor cells and of tumor DNA in plasma before and after tumor resection in rats. Ann. Surg. Oncol..

[19] Bijian K., Mlynarek A.M., Balys R.L., Jie S., Xu Y., Hier M.P., Black M.J., Di Falco M.R., LaBoissiere S., Alaoui-Jamali M.A. (2009). Serum proteomic approach for the identification of serum biomarkers contributed by oral squamous cell carcinoma and host tissue microenvironment. J. Proteome Res..

[20] Liu C.J., Kao S.Y., Tu H.F., Tsai M.M., Chang K.W., Lin S.C. (2010). Increase of microRNA miR-31 level in plasma could be a potential marker of oral cancer. Oral Dis..

[21] Lu C.C., Chang K.W., Chou F.C., Cheng C.Y., Liu C.J. (2007). Association of pretreatment thrombocytosis with disease progression and survival in oral squamous cell carcinoma. Oral Oncol..

[22] Tissot C., Toffart A.C., Villar S., Souquet P.J., Merle P., Moro-Sibilot D., Perol M., Zavadil J., Brambilla C., Olivier M. (2015). Circulating free DNA concentration is an independent prognostic biomarker in lung cancer. Eur. Respir. J..

[23] Huang A., Zhang X., Zhou S.L., Cao Y., Huang X.W., Fan J., Yang X.R., Zhou J. (2016). Plasma Circulating Cell-free DNA Integrity as a Promising Biomarker for Diagnosis and Surveillance in Patients with Hepatocellular Carcinoma. J. Cancer.

[24] Murtaza M., Dawson S.J., Tsui D.W., Gale D., Forshew T., Piskorz A.M., Parkinson C., Chin S.F., Kingsbury Z., Wong A.S. (2013). Non-invasive analysis of acquired resistance to cancer therapy by sequencing of plasma DNA. Nature.

[25] Shukla D., Kale A.D., Hallikerimath S., Yerramalla V., Subbiah V. (2013). Can quantifying free-circulating DNA be a diagnostic and prognostic marker in oral epithelial dysplasia and oral squamous cell carcinoma?. J. Oral Maxillofac. Surg..

[26] Coulet F., Blons H., Cabelguenne A., Lecomte T., Lacourreye O., Brasnu D., Beaune P., Zucman J., Laurent-Puig P. (2000). Detection of plasma tumor DNA in head and neck squamous cell carcinoma by microsatellite typing and p53 mutation analysis. Cancer Res..

[27] Mandel P., Metais P. (1948). Les acides nucléiques du plasma sanguin chez l’homme. C. R. Seances Soc. Biol. Fil..

[28] Leon S.A., Shapiro B., Sklaroff D.M., Yaros M.J. (1977). Free DNA in the serum of cancer patients and the effect of therapy. Cancer Res..

[29] Stroun M., Anker P., Lyautey J., Lederrey C., Maurice P.A. (1987). Isolation and characterization of DNA from the plasma of cancer patients. Eur. J. Cancer Clin. Oncol..

[30] Stroun M., Anker P., Maurice P., Lyautey J., Lederrey C., Beljanski M. (1989). Neoplastic characteristics of the DNA found in the plasma of cancer patients. Oncology.

[31] Tsao S.C., Weiss J., Hudson C., Christophi C., Cebon J., Behren A., Dobrovic A. (2015). Monitoring response to therapy in melanoma by quantifying circulating tumour DNA with droplet digital PCR for BRAF and NRAS mutations. Sci. Rep..

[32] Gautschi O., Bigosch C., Huegli B., Jermann M., Marx A., Chasse E., Ratschiller D., Weder W., Joerger M., Betticher D.C. (2004). Circulating deoxyribonucleic Acid as prognostic marker in non-small-cell lung cancer patients undergoing chemotherapy. J. Clin. Oncol..

[33] Lee G.K., Kim H.Y., Lee J.S. (2011). Circulating cell-free DNA in plasma of never smokers with advanced lung adenocarcinoma receiving gefitinib or standard chemotherapy as first-line therapy. Clin. Cancer Res..

[34] Basnet S., Zhang Z.Y., Liao W.Q., Li S.H., Li P.S., Ge H.Y. (2016). The Prognostic Value of Circulating Cell-Free DNA in Colorectal Cancer: A Meta-Analysis. J. Cancer.

[35] Dawson S.J., Rosenfeld N., Caldas C. (2013). Circulating tumor DNA to monitor metastatic breast cancer. N. Engl. J. Med..

[36] Dawson S.J., Tsui D.W., Murtaza M., Biggs H., Rueda O.M., Chin S.F., Dunning M.J., Gale D., Forshew T., Mahler-Araujo B. (2013). Analysis of circulating tumor DNA to monitor metastatic breast cancer. N. Engl. J. Med..

[37] Desai A., Kallianpur S., Mani A., Tijare M.S., Khan S., Jain M., Mathur V., Ahuja R., Saxena V. (2018). Quantification of circulating plasma cell free DNA fragments in patients with oral cancer and precancer. Gulf J. Oncol..

[38] Ocana A., Diez-Gonzalez L., Garcia-Olmo D.C., Templeton A.J., Vera-Badillo F., Jose Escribano M., Serrano-Heras G., Corrales-Sanchez V., Seruga B., Andres-Pretel F. (2016). Circulating DNA and Survival in Solid Tumors. Cancer Epidemiol. Biomark. Prev..

[39] Cheng J., Tang Q., Cao X., Burwinkel B. (2017). Cell-Free Circulating DNA Integrity Based on Peripheral Blood as a Biomarker for Diagnosis of Cancer: A Systematic Review. Cancer Epidemiol. Biomark. Prev..

[40] Vogelstein B., Kinzler K.W. (1999). Digital PCR. Proc. Natl. Acad. Sci. USA.

[41] Thornton B., Basu C. (2011). Real-time PCR (qPCR) primer design using free online software. Biochem. Mol. Biol. Educ..

[42] El Messaoudi S., Rolet F., Mouliere F., Thierry A.R. (2013). Circulating cell free DNA: Preanalytical considerations. Clin. Chim. Acta.

[43] Moraga D., Panayotova N., Zhou X.H., Farmerie W.G., Shanker S. (2013). Modified Library Construction Method for 3-5kb Illumina Mate-pair Libraries. J. Biomol. Tech..

[44] Serrao E., Cherepanov P., Engelman A.N. (2016). Amplification, Next-generation Sequencing, and Genomic DNA Mapping of Retroviral Integration Sites. J. Vis. Exp..

[45] Nygaard A.D., Holdgaard P.C., Spindler K.L., Pallisgaard N., Jakobsen A. (2014). The correlation between cell-free DNA and tumour burden was estimated by PET/CT in patients with advanced NSCLC. Br. J. Cancer.

[46] Norton S.E., Lechner J.M., Williams T., Fernando M.R. (2013). A stabilizing reagent prevents cell-free DNA contamination by cellular DNA in plasma during blood sample storage and shipping as determined by digital PCR. Clin. Biochem..

[47] Hung E.C., Chiu R.W., Lo Y.M. (2009). Detection of circulating fetal nucleic acids: A review of methods and applications. J. Clin. Pathol..

[48] Chan K.C., Yeung S.W., Lui W.B., Rainer T.H., Lo Y.M. (2005). Effects of preanalytical factors on the molecular size of cell-free DNA in blood. Clin. Chem..

[49] Chan K.C., Zhang J., Hui A.B., Wong N., Lau T.K., Leung T.N., Lo K.W., Huang D.W., Lo Y.M. (2004). Size distributions of maternal and fetal DNA in maternal plasma. Clin. Chem..

[50] Li Y., Zimmermann B., Rusterholz C., Kang A., Holzgreve W., Hahn S. (2004). Size separation of circulatory DNA in maternal plasma permits ready detection of fetal DNA polymorphisms. Clin. Chem..

[51] Kumar S., Guleria R., Singh V., Bharti A.C., Mohan A., Das B.C. (2010). Efficacy of circulating plasma DNA as a diagnostic tool for advanced non-small cell lung cancer and its predictive utility for survival and response to chemotherapy. Lung Cancer.

[52] Guo N., Lou F., Ma Y., Li J., Yang B., Chen W., Ye H., Zhang J.B., Zhao M.Y., Wu W.J. (2016). Circulating tumor DNA detection in lung cancer patients before and after surgery. Sci. Rep..

[53] Ziegler A., Zangemeister-Wittke U., Stahel R.A. (2002). Circulating DNA: A new diagnostic gold mine?. Cancer Treat. Rev..

[54] Agostini M., Enzo M.V., Bedin C., Belardinelli V., Goldin E., Del Bianco P., Maschietto E., D’Angelo E., Izzi L., Saccani A. (2012). Circulating cell-free DNA: A promising marker of regional lymphonode metastasis in breast cancer patients. Cancer Biomark..

[55] Eisenhauer E.A., Therasse P., Bogaerts J., Schwartz L.H., Sargent D., Ford R., Dancey J., Arbuck S., Gwyther S., Mooney M. (2009). New response evaluation criteria in solid tumours: Revised RECIST guideline (version 1.1). Eur. J. Cancer.

[56] Garcia-Olmo D., Garcia-Olmo D.C., Ontanon J., Martinez E. (2000). Horizontal transfer of DNA and the “genometastasis hypothesis”. Blood.

[57] Pinzani P., Salvianti F., Pazzagli M., Orlando C. (2010). Circulating nucleic acids in cancer and pregnancy. Methods.

[58] Valastyan S., Reinhardt F., Benaich N., Calogrias D., Szasz A.M., Wang Z.C., Brock J.E., Richardson A.L., Weinberg R.A. (2009). A pleiotropically acting microRNA, miR-31, inhibits breast cancer metastasis. Cell.

[59] Zhang Y., Guo J., Li D., Xiao B., Miao Y., Jiang Z., Zhuo H. (2010). Down-regulation of miR-31 expression in gastric cancer tissues and its clinical significance. Med. Oncol..

[60] Newman A.M., Bratman S.V., To J., Wynne J.F., Eclov N.C., Modlin L.A., Liu C.L., Neal J.W., Wakelee H.A., Merritt R.E. (2014). An ultrasensitive method for quantitating circulating tumor DNA with broad patient coverage. Nat. Med..

[61] Hussing C., Kampmann M.L., Mogensen H.S., Morling C.B.N. (2015). Comparison of techniques for quantification of next-generation sequencing libraries. Forensic Sci. Int. Genet. Suppl. Ser..

